# The Effects of an In-vehicle Collision Warning System on Older Drivers' On-road Head Movements at Intersections

**DOI:** 10.3389/fpsyg.2021.596278

**Published:** 2021-02-19

**Authors:** Rachel Shichrur, Navah Z. Ratzon, Arava Shoham, Avinoam Borowsky

**Affiliations:** ^1^Occupational Therapy Department, Faculty of Health Sciences, Ariel University, Ariel, Israel; ^2^Occupational Therapy Department, School of Health Professions, Sackler Faculty of Medicine, Tel Aviv University, Tel Aviv-Yafo, Israel; ^3^Occupational Therapy Clinics, Clalit Health Services, Dimona, Israel; ^4^The Department of Industrial Engineering and Management, Ben-Gurion University of the Negev, Be'er-Sheva, Israel

**Keywords:** technology—assistive/supportive, older drivers, in-vehicle camera, feedback, head movements, naturalistic driving

## Abstract

With age might come a decline in crucial driving skills. The effect of a collision warning system (CWS) on older drivers' head movements behavior at intersections was examined.

**Methods:** Twenty-six old-adults, between 55 and 64 years of age, and 16 Older drivers between 65 and 83 years of age, participated in the study. A CWS (Mobileye Inc.) and a front-back in-vehicle camera (IVC) were installed in each of the participants' own vehicles for 6 months. The CWS was utilized to identify unsafe events during naturalistic driving situations, and the IVC was used to capture head direction at intersections. The experimental design was conducted in three phases (baseline, intervention, and carryover), 2 months each. Unsafe events were recorded by the CWS during all phases of the study. In the second phase, the CWS feedback was activated to examine its effect on drivers' head movement' behavior at intersections.

**Results:** Older drivers (65+) drove significantly more hours in total during the intervention phase (*M* = 79.1 h, *SE* = 10) than the baseline phase (*M* = 39.1 h, *SE* = 5.3) and the carryover phase (*M* = 37.7 h, *SE* = 5.4). The study revealed no significant differences between the head movements of older and old-adult drivers at intersections. For intersection on the left direction, a significant improvement in drivers' head movements' behavior was found at T-junctions, turns and four-way intersections from phase 1 to phase 3 (*p* < 0.01), however, two intersection types presented a decrease along the study phases. The head movements' behavior at roundabouts and merges was better at phase 1 compared to phase 3 (*p* < 0.01). There was no significant reduction of the mean number of CWS unsafe events across the study phases.

**Conclusions:** The immediate feedback provided by the CWS was effective in terms of participants' head movements at certain intersections but was harmful in others. However, older drivers drove many more hours during the active feedback phase, implying that they trusted the system. Therefore, in the light of this complex picture, using the technological feedback with older drivers should be followed with an additional mediation or follow-up to ensure safety.

## Introduction

### Older Drivers' Safety

The percentage of older individuals (typically defined as ≥65 years; Vespa et al., 2018; National Highway Traffic Safety Administration, 2020). in society has been steadily increasing worldwide and is expected to reach 90 million in 2050 in only the United States. This population constitutes about a quarter of all licensed drivers (Pomidor, 2015). In 2018, 6,907 drivers above the age of 65 were killed on US roads, constituting 19% of all road fatalities in the US (National Highway Traffic Safety Administration, 2020). In modern life, older adults, similar to all age groups, are dependent on driving as the primary mode of transportation, allowing them to maintain autonomy (Classen, 2010). However, the prevalence of medical impairments, decline in vision, cognition, motor abilities, and somatosensory functions rises with age, which may have substantial effects on driving skills, including the ability to perform proper scanning (Karthaus and Falkenstein, 2016; Samuel et al., 2016).

Researchers suggest that the underlying frailty, medical conditions, and medication-use contribute significantly to crash disparities between older and younger drivers and increased risks of injury and fatality in older drivers (McGwin et al., 2000; Langford and Koppel, 2006). The data of Bédard et al. (2002) showed that older drivers are more vulnerable to the traumatic effects of crashes. The odds of a fatal injury for older drivers (65–79) were 2.3 times of that of drivers aged 40–49 and the odds of a fatal injury for drivers older than 80 was even five times more of that of the younger drivers. Consistently, several studies confirmed that drivers aged ≥65 pose danger to themselves and to other road users as compared with drivers at younger age groups (Dellinger et al., 2004; Awadzi et al., 2008). Findings from a driving simulator study (Park et al., 2017) indicated that older adults (65.6 ± 5 years) have some limitations, primarily relating to left turns against oncoming traffic and while overtaking a lead vehicle. In a study by Bao and Boyle (2009), for example, older drivers (65–80) had a significantly smaller proportion of visual sampling to the left and right-hand side of the intersection during intersection negotiations when compared to younger (18–25) and middle-aged (35–55) drivers.

These circumstances raise the need to balance between encouraging independent living and protecting the rights of the safe older drivers, vs. the practitioners' duty to identify unsafe driving and protect other road users.

### Vision and Safe Driving

Studies agree that for a driver, vision is crucial for collecting driving-relevant information from the driving environment (Van Houten and Retting, 2001; Green, 2002). Visual attention, a critical skill for avoiding crashes while driving, is used to direct information processing resources (using eye and head movements) to spot potentially important visual events. Older drivers (65+) tend to identify hazards less often when hazards are located in the periphery of the visual scene (Bromberg et al., 2012).

Moreover, safe driving relies on the drivers' ability to make quick head turns and eye movements, scan other spatial locations such as mirrors, lead cars, pedestrians, and road traffic signs, and shift their attention to the road. The timing of performing glances before moving forward at an intersection is critical. It takes 1.8–2.9 s to identify approaching vehicles before leaving the stop line (Hostetter et al., 1986 as cited in Fisher et al., 2016). Most drivers make glances to the left and the right; however, only a few make a secondary glance (Fisher et al., 2016). A secondary glance is defined as “a glance toward an area from which a threat might emerge at a time after the foot moves from the brake, or after the start of acceleration into the intersection when there is not a stop” and is considered “the last best chance to abort the movement into the intersection” (Fisher et al., 2016, p. 94).

In this study, head movements were used as a proxy of gaze position. It has already been shown that for horizontal visual angles larger than 30° an observer must move his head toward the target area and the gaze position follow this (Land and Tatler, 2009). In this study the focus was identifying glance position at intersection, which generally requires scanning at large visual angles. In addition, Metz and Krueger (2010) recommend using head movement analysis instead of eye movement at intersections due to severe data loss of eye movements that often occurs when scanning requires wide visual angles that require head movements. The authors added that in their study head movement was a good and reliable alternative to eye movements. Capturing head movements during a drive is becoming more common especially due to the prevalence of low cost in-vehicle cameras that allow it.

### Visual Search for Threats

Visual search is a prominent process that drivers must apply in order to identify road hazards. Visual search evidence in the driving domain shows that scanning patterns are typically different between older and younger-experienced drivers. Bao and Boyle (2009), for example, showed that older drivers do not utilize their full scanning range when compared to middle-aged drivers, and tend to check fewer areas before executing a maneuver through intersections, specifically during left and right turns. Romoser and Fisher (2009), concluded that regardless of driver's cognition, speed-of-processing, or useful field of view (UFOV) status if drivers do not turn their heads to scan for cross-traffic when turning at intersections, they will fail to detect unanticipated vehicles that may conflict with their turn. Inattention can cause drivers to exhibit unsafe behaviors during the driving task, such as greater lane position variability, reduced headway distance, and reduced time-to-collision. Additionally, inattention may also reduce a driver's capability to respond to hazardous situations, as indicated by delayed reactions (Strayer et al., 2003; Lees and Lee, 2007).

### Interventions to Improve Older Drivers' Road Scanning Skills

Despite older drivers deteriorated scanning behavior, some studies have shown that visual search for threats is a skill that can be trained and improved. Romoser and Fisher (2009), for example, used a driving simulator to train older drivers (70+). They found that active training with direct feedback (using a driving simulator), compared to passive training (e.g., telling drivers where to look without actually driving), is a more effective strategy for increasing the likelihood that older drivers will look for threats during a turn. Their active training increased the likelihood of hazard detection during a right or left turn by nearly 100% in both post-training simulator tests and field drives (Romoser and Fisher, 2009). Similarly, Pollatsek et al. (2012) were able to consistently train older drivers (70+) to both scan the roadway environment and to learn to allocate their attention more effectively.

Nevertheless, this evidence shows only an initial step toward finding ways to preserve and even improve older drivers' visual search skills in order to make them safer drivers and compensate for age-related deterioration of driving skill. An important aspect that was overlooked in these studies is the possibility of exploiting the advantages of in-vehicle technology and specifically using collision-warning systems (CWS). This exploitation would be used with the purpose of (1) facilitating more efficient road scanning and (2) providing an overall better safe-driving performance while combining humans with technology vs. humans alone.

Although there currently is encouraging evidence regarding the potential of an intervention aimed to improve older drivers' driving performance at least partially, the way toward making older adults safer drivers is still long. This delay is especially worrying if one considers their over-representation in fatal traffic crashes, poor scanning performance, and overall reduced driving performance compared to younger-experienced drivers. Considering that older drivers look less often to the left and right at intersections than younger adults do, the question then becomes whether there is a way to improve older adults' scanning at intersections by using current in-vehicle technology (Caserta and Abrams, 2007; Bao and Boyle, 2009).

### In-vehicle Data Recorders (IVDR)

In recent years, due to various technological improvements, IVDR technology offers CWS that can help drivers to pay attention to road hazards and objects to avoid collisions (Maltz and Shinar, 2004; Wang et al., 2016; Hubele and Kennedy, 2018). This technology may be especially valuable when other tasks compete for driver's attention. The task of the warning system is to attract the drivers' attention back on to the road, especially when the road demands increase.

Modern in-vehicle safety technologies offer Advanced Driver Assistance Systems (ADAS) that helps drivers drive safer and pay attention to road hazards. ADAS are becoming more ubiquitous in newer cars and may significantly reduce crashes related to impaired visual search, distraction, or lack of attention. Hickman et al. (2015) have collected retrospective crash data from 14 motor carriers including a total of 151,624 truck-years on different types of roads. The authors have demonstrated that lane departure warning (LDW) significantly reduced a LDW-related crashes by 1.92 times and roll stability control (RSC) significantly reduced a RSC-related crashes by 1.56 times. Integrating between real-time on-road driving data, with systems that monitor information regarding the drivers' scanning behavior (such as IVC), ADAS are designated to help drivers identify unnoticed road hazards as well as use the feedback received by the system to facilitate their road scanning behavior training when distracted (Carr and Grover, 2020). Analyzing the different patterns of spatial attention and driving behavior will assist in modifying inferior behaviors in an effort to improve road safety.

Recruiting such technologies to assist the older driver in driving safer, as well as in taking advantage of the feedback received by the system in order to facilitate their scanning behavior, has been set as a goal for current researchers (Carr and Grover, 2020). This research is expected to provide further insight regarding older drivers' spatial attention and head movements behavior in correlation with unsafe driving-related events on the road. Therefore, the present study proposes an intervention procedure that combines CWS and IVC as an integrated tool to enhance older drivers' safety and awareness of safety while driving. The effects of an CWS's feedback on older drivers' unsafe CWS events and head movements will be analyzed.

### Hypotheses

This study has three hypotheses:

1. The feedback-based intervention provided by CWS in phase 2 will be found effective in improving the head movements behavior of study population at intersections and in reducing their involvement in hazardous driving-related events (provided by the CWS).
2. Old-adults group (55–64) will have better head movements at intersections than the older drivers' group (+65).
3. Positive correlations will be found between low quality of the head movements at intersections as measured by IVC and hazardous driving-related events as obtained from CWS in the study phases.

## Methods

The study population included 42 drivers: 26 old-adults (55–64 years old, *M* = 59.4, *SD* = 3.1), and 16 older drivers (65–83 years old, *M* = 70.9, *SD* = 5.32). The study population included 25 men and 17 women. All participants were independent drivers with a valid driver's license and with normal or corrected to normal vision. Recruitment was done via e-mails, and snowball sampling method, targeting old-aged volunteers who pursue an independent lifestyle. Volunteers were compensated for the time they spent participating in the study for 6 months.

To exclude potential effects of depression and cognitive impairments, participants had to score five or fewer points on the Geriatric Depression Scale (Yesavage et al., 1982) and to score above 24 in the Mini-Mental State Examination (Folstein et al., 1975). Other exclusion criteria included a medical history of neurological, orthopedic, and/or psychiatric conditions with permanent impairments or using drugs that, according to the guidelines of the pharmaceutical company, may interfere with driving. In each participant's private vehicle, a CWS and IVC were installed to identify unsafe events during on-road driving and to capture head movements' behavior data at intersections.

### Primary Outcome Measure Tools

#### Collision-Warning Systems (CWS)

ADAS technology offers collision-warning systems (CWS) that provide a forward collision warning (FCW) and a lane departure warning (LDW), helping drivers drive safely. The CWS is a vision-based tool for the vehicle, which continually measures the time/distance to the vehicle in front and lane marks. The ultra-speed data processor includes algorithms that follow lane marks and road curves, detect cars and pedestrians, and follow the headway distance, working in the rain and at night. It is programmed to detect only five types of objects, specifically: trucks, cars, motorcycle, bicycles, and people. The system follows four types of safety outcomes.

(a) *Urban Forward Collision Warning (uFCW)*—A risk warning for urban collision is activated when driving is <30 km/h. The system calculates the time it will take to stop the car without touching the other car, and a risk event is recorded when the distance between the cars is 2.7 s.

(b) *Forward Collision Warning (FCW)*—A risk warning for rear-end collision is activated when driving 30 km/h and above. The system calculates the time it will take to stop the car without touching the other car, and a risk event is recorded when the distance between the cars is 2.7 s.

(c) *Unsafe Headway Warning (HW)***—**The warning is activated from 30 km/h and above. The system calculates the time it will take to stop the car without colliding with the object in front of the vehicle, and a risk event is recorded when the distance between the car and one of the aforementioned objects is 1 s.

(d) *Sudden Lane Deviations Warning (LDW)*—A lane departure without signaling warning is activated from 55 km/h and above. The CWS focuses on driving safety and analysis of the technical skill of the driver and provides a driving profile. The riskiness grade used in this study obtained by the CWS is a calculation of the mean number of all types of unsafe events per hour from the four types of safety outcomes (uFCW, FCW, HW, and LDW).

#### In-vehicle Front-Back Camera (IVC)

Each vehicle was equipped with a dual-lens video camera that captured both the driving scene from the driver's perspective (i.e., driving context) as well as the driver's face. This front-back camera configuration allowed capturing the participant's head direction dependent on the driving context (e.g., driving on a straight road, approaching an intersection). The camera had a high definition (HD) video quality and a 64 GB SD card to allow recording 9 h of driving each time. Once the card was full, it was removed from the vehicle, analyzed on a computer, and replaced with a blank card. Recording the driver's face provided the information regarding head movements.

### Data Preparation

**Figure 1:**
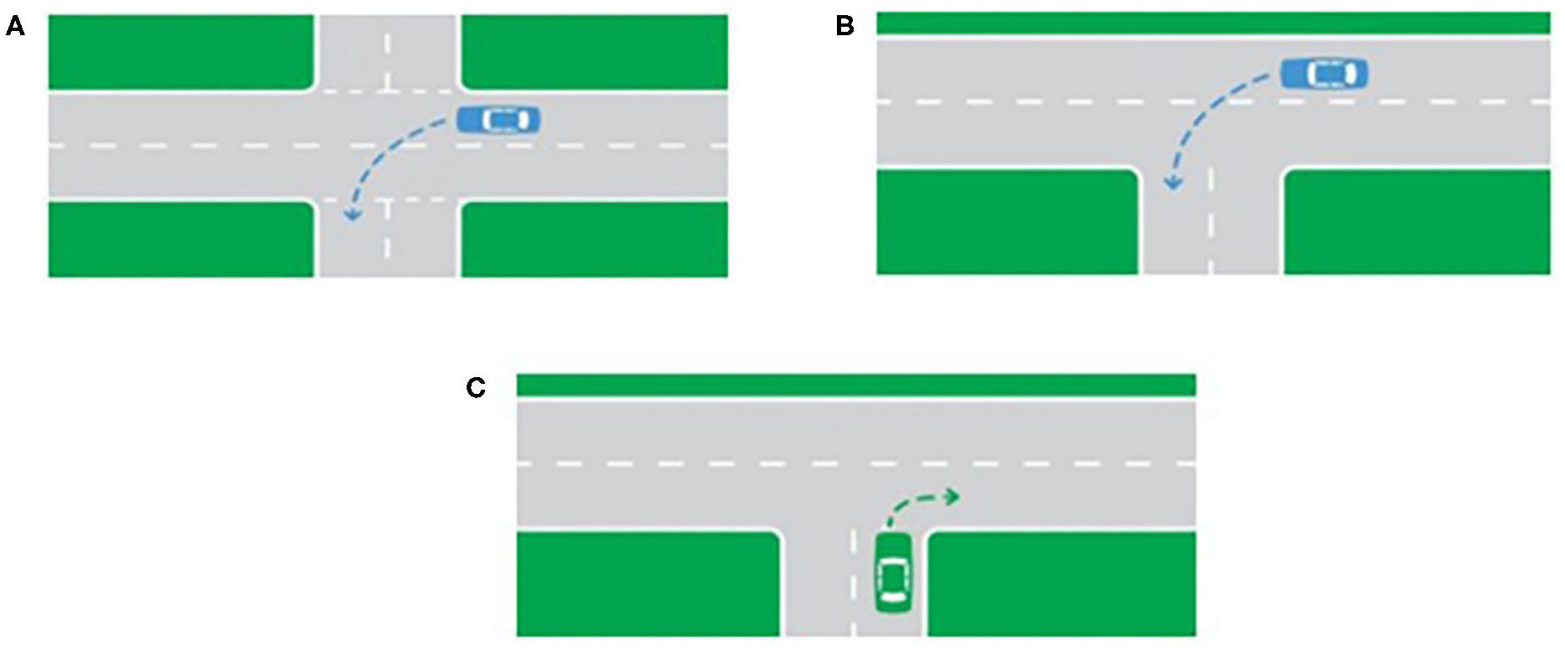
**(A)** A left turn at a four-way intersection. **(B)** A left turn at a two-lane T junction. **(C)** A right turn at a T junction.

As mentioned above, the front-facing camera provided an immense amount of real-world driving data of the traffic environment from a driver's perspective. Thus, at each phase of the study, it was decided to focus on the first 120 intersections that each participant encountered and examine the head movement behavior of each participant at each intersection. The intersections were classified into five types according to their geometric structure (merging road, roundabout, turn, T junction, and four-way intersection). The direction of travel of the driver (right/left/straight and also the presence of secondary glances when needed) and the presence of other road users (vehicles and pedestrians) were registered. Examples of different types of intersections can be seen in Figures 1A–C.

### Procedure for Coding Research Data

Each coder was given a list of proper road scanning behavior expected at each intersection that was predefined by the research team [a complete list of all head movements demands for each intersection is provided in Appendix A (Supplementary Material)]. Each coder was asked to indicate whether the driver scanned the intersection properly (given a score of “2”), whether the road scanning behavior was only partially correct (given a score of “1”), or whether the scanning behavior at that specific intersection was improper (given a score of “0”) for each intersection per participant. In cases where a coder was not sure how to classify the driver's head movement behavior at a specific intersection, he or she consulted with the research team, who made a classification decision based on a discussion.

Primary, a pilot data-coding procedure was conducted where four researchers from the research team analyzed two drivers' videos. Each researcher viewed the camera videos independently, identified 30 intersections, classified them, defined the head movements requirements at each intersection, and reviewed the actual head-based behavior of each driver. A between rater reliability test of this pilot data coding procedure showed reliability of only 76.6%. In order to improve the inter-rater reliability percentage, three additional meetings were required in which the researchers consulted on uniformity in interpreting the data obtained from the videos. This process was repeated until the raters were able to achieve inter-rater reliability of over 90%. To promote the research, three additional research assistants, trained by the research team, were recruited to view camera videos and code the participants' horizontal head rotations. Each research assistant viewed the same 30 intersections of the same driver, and classified his head movements as: proper head movements = “2,” partially proper head movements = “1,” or improper head movements = “0.” Checking the inter-rater reliability of the three research assistants in analyzing the same 30 intersections showed high reliability of 97%.

### Experimental Design

The experimental design was a *A2*<P3^*^I5^*^D4> mixed design. The between-subjects independent variables included the age group (A: old-adults or older drivers). The within-subjects independent variables included the experiment's phase (P: 1, 2, or 3), the intersection type (I: 1–5, see data preparation section), and the travel direction (D: 1–4, see data preparation section). The dependent variables included the score of the head movements (proper head movements = “2,” partially proper head movements = “1,” or improper head movements = “0”), the number of unsafe events per hour for each one of the four types of safety outcomes (uFCW, FCW, HW, and LDW), and CWS riskiness grade (Mobileye, Inc.).

### Procedure

The institutional review at Tel Aviv University board approved this study. Informed consent forms were obtained from all the volunteers prior to commencing the study. After signing the informed consent, each participant completed the questionnaires that were relevant for the screening tools, and only those who met the inclusion criteria were able to participate in the study. Since this study is focused on naturalistic driving, the research location was determined according to the participants' convenience in their community (e.g., a quiet room in a community center or the driving laboratory at the University). Next, the CWS was installed by expert technicians in each of the participants' own vehicle at his/her house. After verifying the system functioned properly, each driver was given a personal identification code number to type in before starting the vehicle. The technicians disassembled the systems after a period of 6 months, at the end of the study.

The study included three phases, and each phase lasted about 2 months. During the first (silent) phase, unsafe events were recorded without the use of active alerts. In the second phase (intervention), the CWS feedback was activated in all the participants' vehicles to examine its effectiveness. In the third phase, the feedback was silenced to examine behavioral change. In each phase, the drivers' head movements were also examined. A comparison was made between the three phases of the study for the two age groups, and also each subject was self-compared between the pre- and post-intervention phase. The study period included a variety of driving events (such as lane deviations and risk of rear-end collisions), under a variety of road or traffic situations (such as dense traffic in urban roads or inter-urban highways) and weather conditions.

### Data Analysis

In order to assess the effectiveness of the feedback-based intervention at improving head movements' behavior, several statistical tests were carried out within the Generalized Linear Mixed Models (GLMM) framework. The head movements behavior analysis, included a logistic regression model with a logit link function. The independent variables that were included in the initial model included: age group (old-adults and older drivers), gender, phase (1, 2, or 3), and intersection type (1–5). The initial model also included three second-order interactions of intersection type^*^phase, age group^*^phase, and intersection type^*^age group, and a third-order interaction of phase^*^age group^*^intersection type. Participants were also included into the model as a random effect. The dependent variable included head movements' behavior that was coded as a binary variable (proper head movements behavior = 1 and Improper head movements behavior = 0). Notably, although we initially coded partially proper behavior as well, due to the small number of cases they were not included in the analysis. This logistic regression model was applied twice: once for the left travel direction and once for the right travel direction. A backwards elimination procedure was applied for each model such that non-significant interactions were removed in case they were not statistically significant.

In order to statistically examine the effect of the feedback-based intervention in reducing risk-related driving events we applied a repeated measures analysis within the GLM framework in SPSS. This model was applied twice. One model included the average number of CWS events per km driven per participant as the dependent variable and the other model included the average number of CWS events per hour driven per participant. Phase was included as the independent variable in both models.

In addition, Pearson correlations were used to examine the correlations between head movements behavior and unsafe driving events. Analysis of the research videos revealed that drivers behaved differently at traffic light intersections as opposed to unsignalized intersections because they were guided by the light signals and did not have to scan the environment themselves. Thus, signalized intersections were excluded from the study. As a result, the percentage of four-ways intersections and T junctions was low relative to other intersections in the sample as in Israel, traffic lights control most In order to statistically examine driving characteristics differences between the two age groups and across the three phases of the study, two linear regression models were applied within the framework of General Linear Model (GLM). This GLM was applied twice: once for number of kilometers driven and once for number of hours driven as dependent variables. The independent variables that were included in the initial models were: phase (1, 2, or 3), and age group (old-adults and older drivers). The models included a second-order interaction of age group^*^phase. Participants were included as a random effect of these types of intersections.

All analyses were carried out at a significant level of 5 percent. Next, all significant effects of the final model were further analyzed using *post-hoc* pairwise contrast comparisons analysis where the Bonferroni correction procedure for multiple comparisons was also applied whenever it was required.

## Results

The results chapter includes four main sections. The first section presents an examination of the feedback-based intervention efficacy on the study population (hypothesis 1). The second section presents the analyses related to the participants' head movements at intersections and differences between age groups (hypothesis 2). The third section presents the correlations between head movements at intersections and unsafe events (CWS) (hypothesis 3). Section four presents additional analyses related to driving exposure in terms of hours driven and distance traveled.

### Section 1: Examining the Efficacy of the Feedback-Based Intervention on the Study Population

Hypothesis 1 estimated that the feedback-based intervention provided in phase 2 by the CWS will be found effective in improving the head movements of study population at intersections. In order to assess the effectiveness of the feedback-based intervention at improving head movements' behavior and reducing unsafe driving events, the study used a linear regression within the GLMM framework. As presented in Table 1, the phase variable was found significant [*F*_(2,3,638)_ = 4.26, *p* < 0.01]. Table 1 presents a summary of the significant fixed effects that were included in the final model.

**Table 1:** A summary of the final logistic regression model's fixed effects of head movements.

**Fixed variables**	***F***	**df2**	**df1**	**sig**
Corrected model	46.9	3,638	16	0.00
Age group	0.03	3,638	1	0.86
Gender	0.37	3,638	1	0.54
Phase	4.26	3,638	2	0.01
Intersection type	135.7	3,638	4	0.00
Intersection type^*^phase	7.9	3,638	8	0.00

* p < 0.05, **p < 0.01.

**Figure 2:**
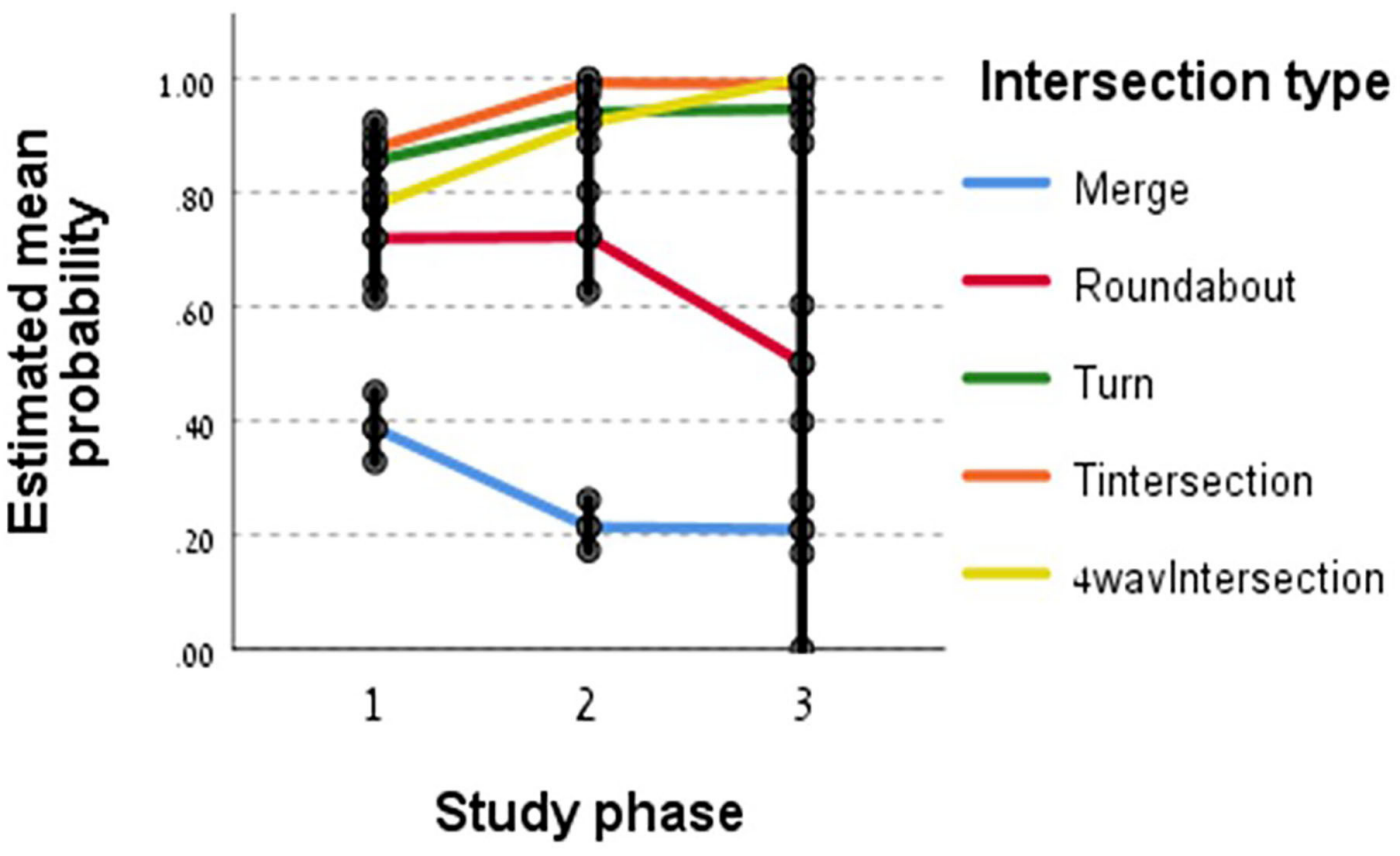
Interaction between intersection types and study phases.

The final regression model for examining the head movements showed that the independent variables entered into the model found to be significant were phase, intersection type and their interaction. *Post-hoc* pairwise contrast comparisons of the significant interaction between the intersection type and the phases of the study are shown in Figure 2. The Y axis presents the estimated mean probability of proper head movements at intersections.

According to Figure 2, T-junctions, turns and four-way intersections drivers' head movements' behavior was better at phases 2 and 3 compared to phase 1 (*p* < 0.01). A significant difference was found between phases 1 and 3 (*p* < 0.01). There was no significant difference between phases 2 and 3 for these three types of intersections. However, two intersection types presented a decrease along the study phases. Roundabout and merges head movements' behavior was better at phase 1 compared to phase 3 (*p* < 0.01). At merges the decrease from phase 1 to phase 2 was significant (*p* < 0.01) but there was no significant difference between phases 2 and 3 and for roundabout there was no significant difference between phases 1 and 2 but the decrease from phase 2 to phase 3 was significant (*p* < 0.01).

**Figure 3:**
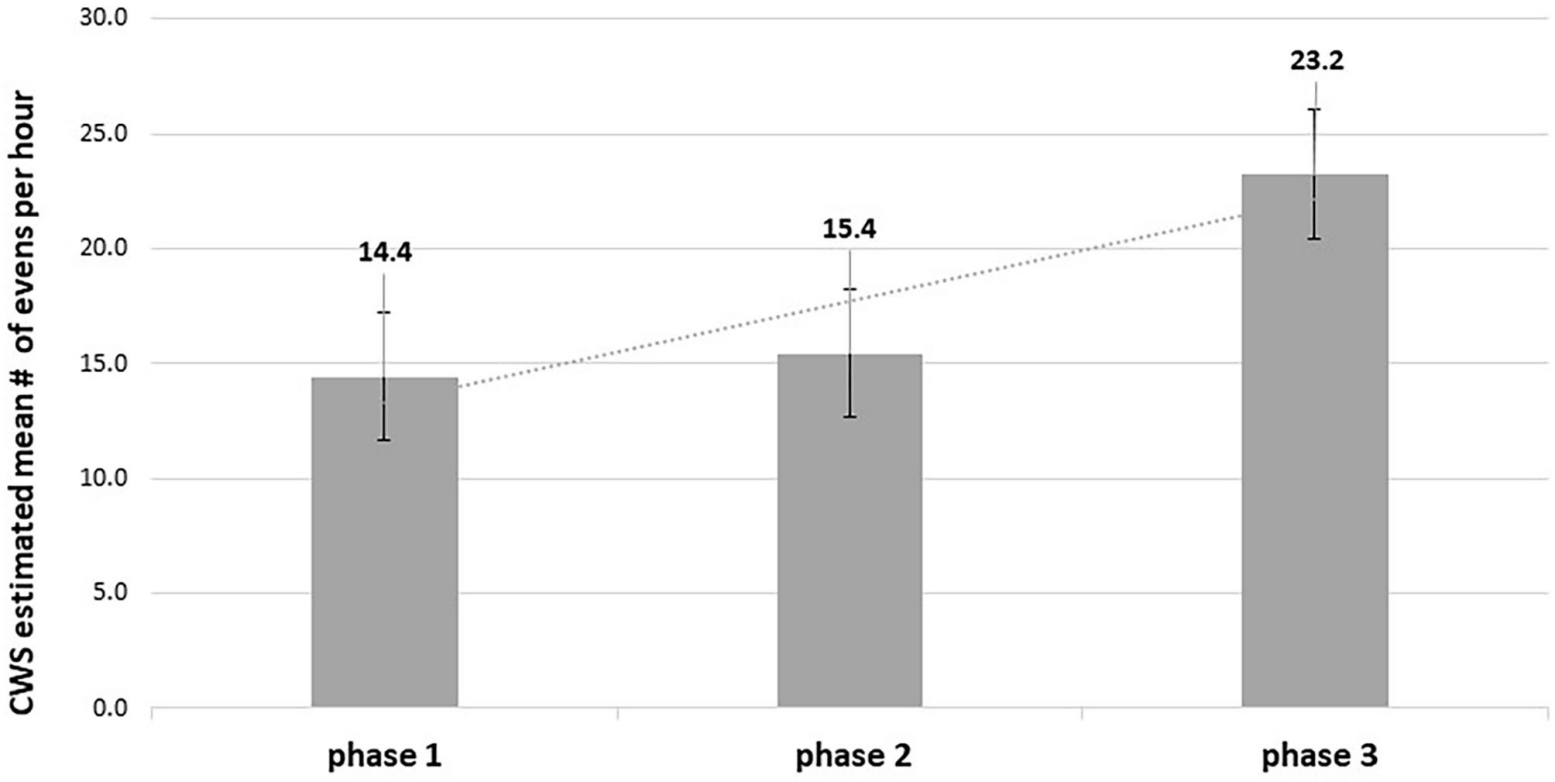
CWS estimated mean number of events per hour throughout the study phases (*n* = 40).

In order to examine the effect of the feedback-based intervention in reducing risk-related driving events as mentioned in hypothesis 1, a repeated measures analysis within the GLM framework was applied. Both models didn't present any significant differences between the average number of CWS events (neither the model per hour nor the model per km driven) per participant. Figure 3 illustrates driving patterns according to CWS unsafe events per hour before, during, and after the intervention.

The CWS mean number of unsafe events per hour refers to the whole drivers' population. According to Figure 3 although the mean number of safety incidents per hour is noticeably increasing across the study' phases, there were no significant differences between the mean number of CWS unsafe events per hour across the phases of the study.

### Section 2: Analysis of Head Movements' Behavior at Intersections

As noted in the method section, the dependent variable representing the quality of the head movements' behavior was coded into three levels: proper head movements' behavior = “2,” partially proper head movements' behavior = “1,” and improper head movements' behavior = “0.” The Percentage of improper, partially proper and proper head movements were computed for each type of intersection (5 types altogether). The descriptive information regarding improper head movements' behavior is presented in Table 2.

**Table 2:** Mean proportion (%) of improper head movements' cases at intersections in each of the study phases.

**Phase**	**M-R**	**M-L**	**Ro-F**	**Ro-R**	**Ro-L**	**Tu-R**	**Tu-L**	**Tj-R**	**Tj-L**	**4W-F**	**4W-R**	**4W-L**
1	30	30	5	1	9	2	0	0	1	22	0	0
2	28	25	3	4	12	3	3	0	0	21	1	0
3	31	26	4	1	13	3	2	0	0	20	0	0

*R, Right; L, Left; F, Forward; M, Merge; Ro, Roundabout; Tu, Turn; Tj, T-junction; 4W, 4-ways intersection*.

*This table is based on 120 junctions per participants. The mean proportions are based on 40 participants in phase 1, 36 participants in phase 2, and 37 participants in phase 3. The sum of each row in this table equals 100%*.

As showed in Table 2, most improper head movements at intersections were associated with right or left merges, forward four-ways intersection, and roundabout to the left across the study phases.

Hypothesis 2 estimated that the old-adults group (55–64) will have better head movements at intersections than the older drivers group (+65). The purpose of the current analysis was to examine this assumption. In order to test whether the differences in the head movements' behavior between the old-adults and the older drivers are significant across the three study's phases, we used a logistic regression model within the GLMM framework as noted in the data analysis section. Notably, since the model of all 5 intersection types at the right direction did not yield significant effects, it is not presented here, and the study focuses on left direction only. As shown in Table 1, the final logistic regression model revealed that no effect was statistically significant for the age group and its interactions with the study phase or intersection type. In other words, there were no significant differences between the mean percentage of head movements' behavior of older and old-adult drivers at intersections across the three phases of the study, suggesting that hypothesis 2 was rejected.

### Section 3: Correlations Between Head Movements at Intersections and Unsafe Events

Hypothesis 3 anticipated positive correlations between poor head movements' behavior at intersections as measured by IVC and hazardous driving-related events as obtained from CWS across the study phases. This analysis was aimed at examining whether the quality of the head movements' behavior at intersections was related to the number of risky events produced by the CWS. Table 3 presents the correlations between head movements and the number of unsafe events. The CSW in the table represents the riskiness grade obtained by the total mean number of all unsafe events per hour.

**Table 3:** Correlations between head movements and unsafe driving.

**Phase**	**Head movements**	**CWS^a^**	**HW^b^**	**uFCW^c^**	**LDW^d^ (right)**	**LDW^d^ (left)**
1	Improper	−0.01	−0.03	−0.08	0.05	0.002
	Partially proper	−0.07	−0.04	0.05	−0.10	−0.03
	Proper	0.02	0.03	0.04	−0.01	0.01
2	Improper	0.06	0.00	−0.09	0.32	−0.07
	Partially proper	0.48^**^	0.38^*^	0.15	0.36^*^	0.56^**^
	Proper	−0.19	−0.11	0.06	–0.41^*^	−0.08
3	Improper	0.20	−0.07	0.32	0.26	0.38^*^
	Partially proper	0.29	0.26	−0.08	0.38^*^	−0.06
	Proper	–0.43^*^	−0.15	−0.26	–0.54^**^	−0.31

* p < 0.05 and

** *p < 0.01*.

a *Collision warning system (riskiness grade)*.

b *Headway warning*.

c *Urban forward collision warning*.

d *Lane departure warning*.

Through examining the correlations between head movements' behavior and risky driving events, it seems that the statistically significant correlations are mainly found in phases 2 and 3. Moderate positive correlations were found for the improper head movements and negative correlations for the proper head movements at all CWS categories. The positive correlations indicate that as the percentage of the improper head movements increases, the number of unsafe events increases as well. Diversely, the significant negative correlations indicate that when the proper head movements rate increases, the number of unsafe events decreases.

### Section 4: Driving Exposure (Total Number of Hours Driven and Distance Traveled)

**Figure 4:**
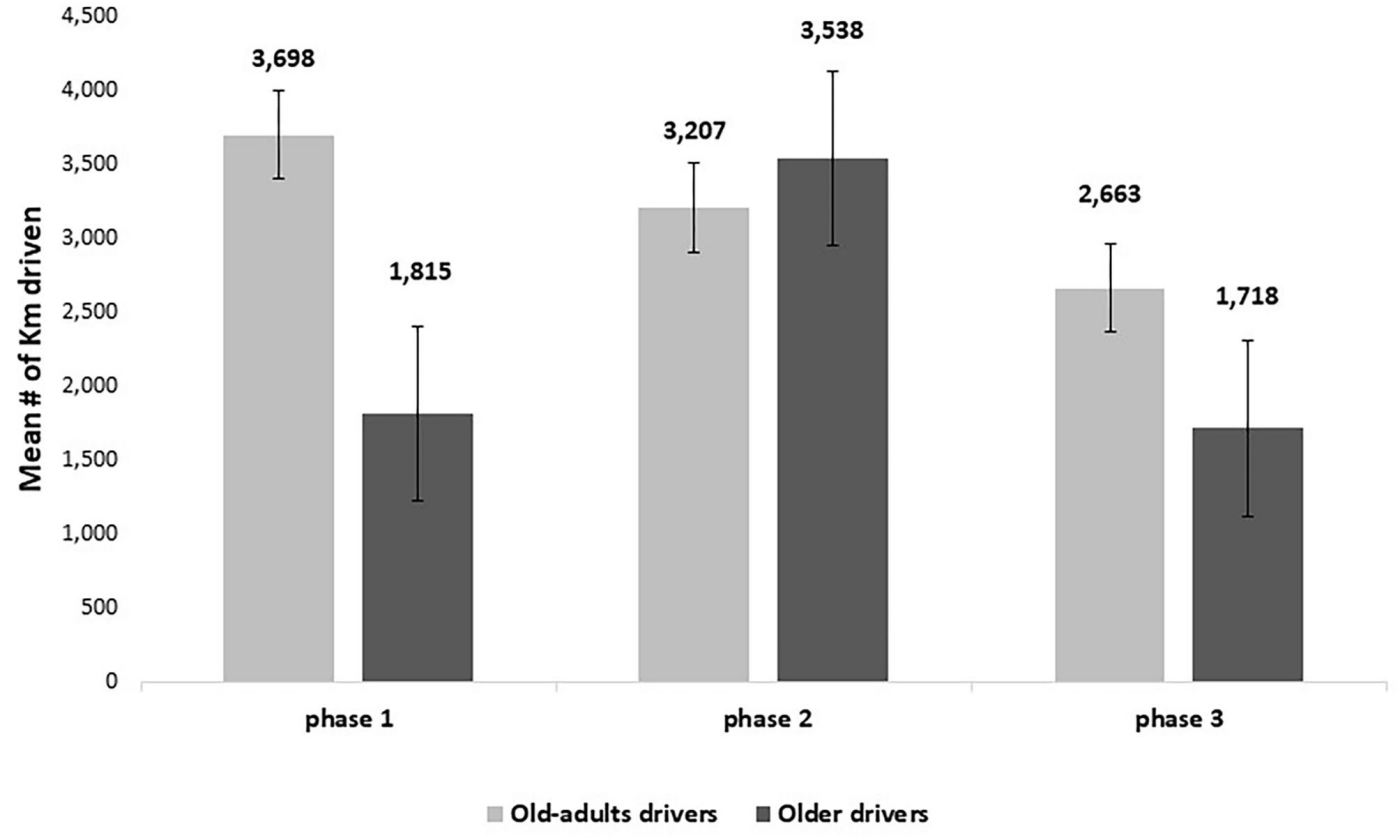
Mean number of kilometers driven per age group.

Additional analyses were carried out to examine the differences between driving exposure of old adults and older drivers. The two age groups that participated in the study, old adults and older drivers, did not significantly differ in terms of gender and education variables as well as in driving history-related variables such as driving days per week and road accident history. The average age was different between the groups consistent with the experimental design. During the entire study period (6 months), the old-adults group drove a total of 9,568 km, while the older group drove a total of 7,071 km, however, the difference was not statistically significant. The final GLM model included one significant main effect of phase [*F*_(2,68)_ = 5.957, *p* < 0.01] and one significant second-order interaction of age group^*^phase [*F*_(2,68)_ = 5.695, *p* < 0.01]. Figure 4 presents the average total number of kilometers driven by each group per phase (i.e., along a period of 2 months per phase).

Phases 1–3 represent the pre-intervention, intervention, and post-intervention phases, respectively. According to Figure 4, the older drivers appeared to travel a shorter distance than the old-adult drivers in phases 1 and 3. However, during the intervention phase, where the feedback from the CWS was activated, they traveled a more considerable distance than the old-adult drivers. *Post-hoc* pairwise contrast comparisons using the Bonferroni correction for multiple comparisons revealed that among the older drivers, phase 1 (*EM* = 1815.3 km, *SE* = 286.4) was not significantly different from phase 2 or 3 (*EM* = 3537.7 km, *SE* = 520.6; *EM* = 1717.6 km, *SE* = 385.8, for phase 2 and 3, respectively). Phase 2 was significantly different from phase 3, suggesting that drivers tended to drive many more kilometers during the intervention phase than the post-intervention phase. Among the old-adults, there was a significant difference between the total number of kilometers driven in phases 1 (*EM* = 3697.7 km, *SE* = 602) and 3 (*EM* = 2662.9 km, *SE* = 438.9). However, the intervention phase (*EM* = 3207 km, *SE* = 455.7) was not statistically significant in either phase 1 or phase 3.

**Figure 5:**
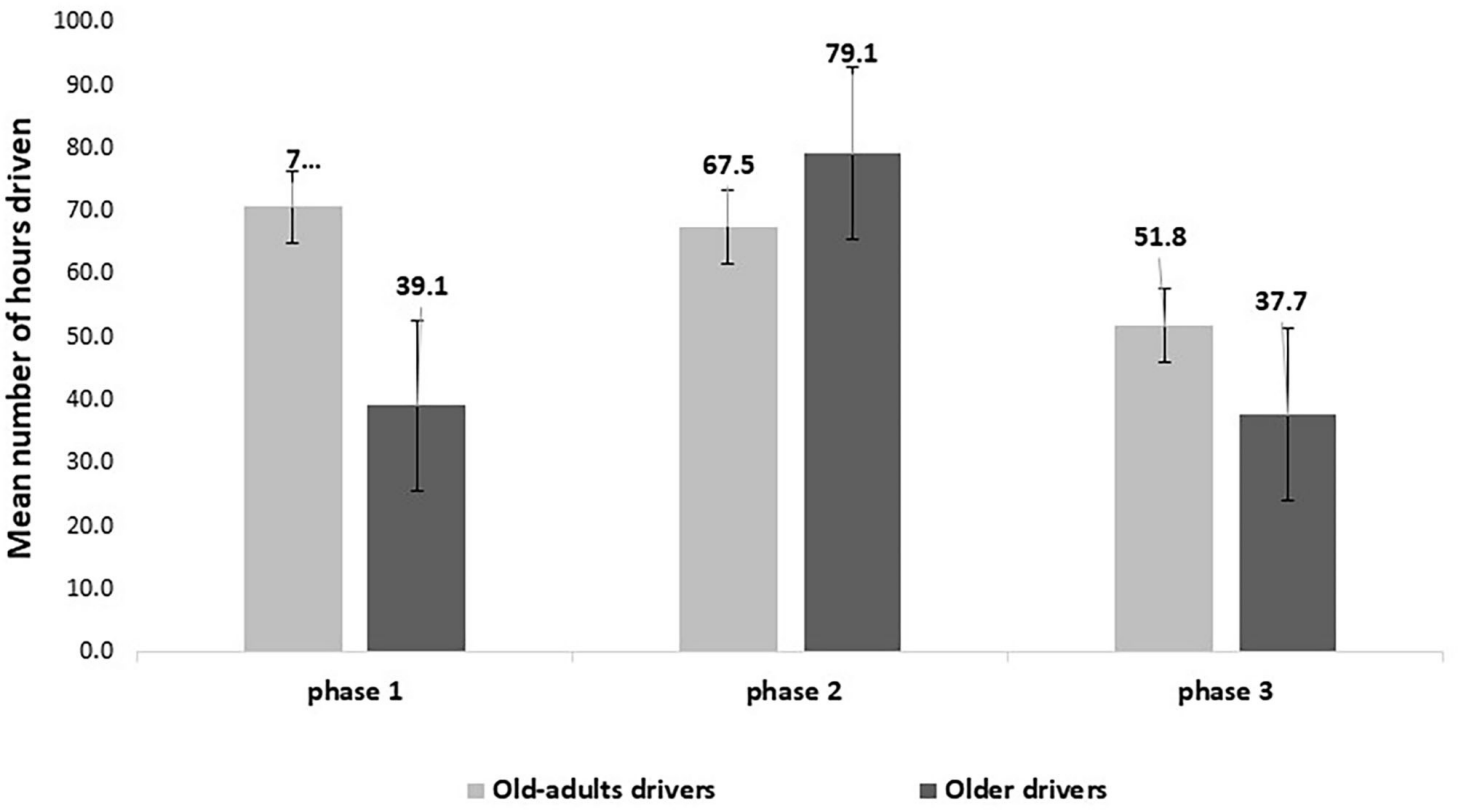
Mean number of hours driven per age group.

To further characterize the participants' driving patterns, the study also examined the total number of actual driving hours, defined as the number of hours driving after reducing the total number of standing engine hours (when the engine is activated without driving). The study found that the old-adult group drove a total of ~190 h, while the older drivers' group drove in total ~156 h. The GLM model included one significant main effect of phase [*F*_(2,70)_ = 7.428, *p* < 0.01] and one significant second-order interaction between age group^*^phase [*F*_(2,70)_ = 4.03, *p* < 0.05]. Figure 5 presents the total number of hours driven for each phase of the study.

Phases 1–3 represent the pre-intervention, intervention and post-intervention phases, respectively. According to Figure 5, the older drivers appeared to be driving fewer hours than the old-adults in phases 1 and 3. However, during the intervention phase, where feedback from the CWS was activated, they drove more hours than the old-adults. *Post-hoc* pairwise contrast comparisons of the main effect revealed that the older group phase 1 (*EM* = 39.1 h, *SE* = 5.3) was not significantly different from phase 3 (*EM* = 37.7, *SE* = 5.4). However, phase 2 (*EM* = 79.1, *SE* = 10.0) was significantly different from phase 1 and 3, suggesting that drivers tended to drive many more hours during the intervention phase than the post-intervention phase. Among old-adults, there was a significant difference between the total number of hours driven in phases 1 (*M* = 70.6, *SE* = 10.8) and 3 (*M* = 51.8, *SE* = 8.7), with a smaller total number of hours driven in the post-intervention phase compared to the pre-intervention phase. There was no significant difference between the intervention phase (*M* = 67.5, *SE* = 9.8), and phases 1 and 3.

## Discussion

The present study was aimed at examining the influence of a collision warning system (CWS) visual or auditory feedback on older drivers' driving and head movements' behavior. The feedback-based intervention provided by CWS was found to be effective in improving study population's head movements' behavior at certain intersections such as T-junctions, turns and four-way intersections but was not effective at roundabout and merges, on the left direction.

The study findings showed that most of the improper head movements were associated with the types of intersections such as right or left merges, turning left at roundabouts and forward four-ways intersection. Possible explanations are divided between drivers' real road scanning difficulties or problems arising from excessively strict coding of improper head movements at these intersection types. At merges for example, it is possible that drivers were not scanning properly side roads that merge from the left because they knew they had the right-of-way forward and believed that the “responsibility” of road scanning was on the merging drivers.

Analyzing the IVC research videos showed that drivers rarely turn their heads sideways at road merges and therefore “gained” a high percentage of improper head movements. In the study of Lemonnier et al. (2020), they found most of the significant effects on oculomotor behaviors, but not on head orienting behaviors. In the same way, this may partly explain the high score of improper head movements in this specific type of intersection. Little is known about drivers' scanning behavior of merging side roads because most studies that measure drivers' road scanning behavior rarely focus on merges of side roads into the main road (Cheng et al., 2016). Moreover, the very few studies that investigated road scanning patterns at merging side roads focused on the scanning patterns of drivers who were merging into the main road rather than on drivers who were driving on the main road.

The current study also demonstrates that the proper head movement's behavior at roundabouts deteriorated from phase 1 and 2 to phase 3. We assume that the reason for the lack of effectiveness lies in the characteristics of the CWS feedback. The CWS feedback is not very effective at low speeds to begin with and most of the safety warning are activated only when driving speed is above 30 or 55 km/h (including FCW, HW, and LDW). Due to the fact that roundabouts are a known traffic calming countermeasure where lower speeds are generally observed (Zubaidi et al., 2020), the warnings were probably less active than the usual. Roundabouts are considered in the literature a good alternative to signalized or controlled intersections because of several advantages, such as the reduction of speed and fatal crashes and the enhancing of traffic capacity (Zubaidi et al., 2020). However, despite these advantages that roundabouts provide, it seems that crashes still occur. The recent study of Zubaidi et al. (2020) investigating the factors that contribute to injury severity sustained by drivers involved in crashes at roundabouts, found that one of the contributory factors was that vehicles did not wait to make a left turn. The current study findings are consistent with this research demonstrating a high percentage of improper head movements when turning left at the roundabouts. When turning left, the operational characteristics of roundabouts every time traffic approaches an entry point, should force drivers to slow down and regard the traffic entering the roundabout otherwise a possible conflict between road users could occur.

The study findings, showing a high percentage of improper head movements at four-ways intersections without a traffic light during the first phase, justify the global tendency to transform them to be controlled intersections or roundabouts (Zubaidi et al., 2020). The complex geometric structure of uncontrolled four-ways intersections can explain the high percentage of improper head movement behavior scores at phase 1 and the improvement throughout the phases as drivers needed the CWS intervention to assist their road scan behavior. According to the literature, four-ways intersections require extra visual scanning that includes looking in both directions and, in addition, performing a secondary scan by making another glance (Romoser and Fisher, 2009; Yamani et al., 2015; Samuel et al., 2016). In a review article by Samuel et al. (2016), they emphasize the importance and complexity of taking secondary glances at these types of intersections. Further research is needed to analyze the difficulties of older drivers in performing secondary glances at intersections using IVC and CWS to develop scanning behavior interventions, improve road scanning ability and thus reduce the risk of car accidents in older drivers (Romoser and Fisher, 2009; Samuel et al., 2016; Lococo et al., 2018).

Findings of the study revealed that the intervention phase improved the road scanning head movements of the study population at T-junctions and turns. According to Bao and Boyle (2009), older drivers do not utilize their full scanning range when compared to middle-aged drivers, and tend to check fewer areas before executing a maneuver through intersections, specifically during left and right turns. Similar to the results of simulator intervention studies (Romoser and Fisher, 2009; Pollatsek et al., 2012), the results of the current naturalistic study demonstrated evidence of improved older drivers' head search behavior at these specific intersections, considered dangerous intersections, with the advantages of using CWS technology.

In opposed to hypothesis 1, the intervention was not found to be effective in reducing drivers' involvement in hazardous driving-related events. The current study findings seem to contradict the conclusions of several studies that have investigated the effects of immediate feedback on improving driving performance by examining unsafe events measured by IVDR (Campbell et al., 2007; Toledo et al., 2008). Also, studies investigating the effectiveness of IVDR's immediate feedback in young drivers have shown significant improvement in speeding and additional non-significant improvement in acceleration and hard braking (Farmer et al., 2010; Farah et al., 2014). A retrospective study by Hickman et al. (2015) shows improvement in truck fleets following the introduction of the LDW system. These studies, however, investigated relatively young drivers (teens and young adults) compared to the current study older population. In fact, the current studies' results are in alliance with some of the recent studies concerning older drivers. Only one study was conducted on older Japanese taxi drivers and CWS. It was published only in the Mobileye's website^1^ and this research concludes that once the CWS was installed, the number of accidents due to front-end collisions decreased by 85% down to zero and also significantly improved driving habits. A close examination of the Japanese study, reveals that they combined the feedback of collision avoidance systems with robust driver training by the fleet managers to improve driver's behavior further. It was found that although advanced safety features and automated vehicles offer great potential to improve road safety and the mobility of drivers, older drivers are skeptical about this technology and are least likely to rely on ADAS to improve their safety on the road (Robertson et al., 2017; Nielsen and Haustein, 2018).

Researches claim that the reason immediate negative feedback is not sufficiently effective over time is that drivers forget most of their near-accidents very quickly, and therefore it is worthwhile that drivers will be given retrospective feedback in addition to immediate feedback (Chapman et al., 2000). A retrospective feedback shows drivers a summary of their driving patterns to raise their awareness and motivation and make a real change in behavior for more extended time periods (Chapman et al., 2000). This argument is supported by findings from other domains such as gamification. Xie (2016), for example, have shown that when young drivers are driving in a driving simulator and receive meaningful feedback with game-design elements, it results in reduced distraction and motivates lasting behavioral changes. Two studies examined the long effects of retrospective feedback, which was conducted through tracking on a dedicated web site for drivers use, indicated an immediate improvement from the beginning of the study. In one study, the improvement was maintained throughout the 9 months of the driving monitoring (Musicant et al., 2007). In the second study, the improvement was maintained over a 4-month period, while in the fifth month, an increase in the number of undesired events above the baseline average was evidenced (Lotan and Toledo, 2006). Intervention studies from various fields have demonstrated the importance of active professional mediation for successful intervention and achieving significant improvement (Lahav et al., 2008; Ratzon et al., 2009; Romoser and Fisher, 2009). The CWS is currently considered a “Stand-Alone” technology (meaning only the driver receives the technological feedback), and it is not enough to be used as an educational tool for older drivers. Therefore, additional mediation or follow-up is recommended for ensuring older drivers' safety.

In contrary to Hypothesis 2, the results of the study showed that there were no significant differences between old-adults and older drivers in all types of head movement's behavior—mainly proper and improper head movements throughout the three phases of the study. These findings are not in accordance with the literature. According to several studies, it has been shown that older drivers perform a reduced road scanning comparing to old-adults or experienced young drivers while driving at intersections (Bao and Boyle, 2009; Dukic and Broberg, 2012; Romoser et al., 2013). However, the present study compares older drivers to old-adults, aged 55–65, and maybe if the study had compared older drivers to younger drivers, the result would have been more similar to literature.

Visual distraction is considered among the major causes of road accidents (Khan and Lee, 2019). Therefore, the current study examined the relationship between head movements' behavior and risky driving. Analyzing the correlations between CWS unsafe events and head movements' behavior revealed that Hypothesis 3 was verified. It was found that mainly in phases 2 and 3, there were statistically significant moderate positive correlations between improper head movements' behavior at intersections as measured by IVC and hazardous driving-related events as obtained from CWS. Additionally, there were significant negative correlations with proper head movements' behavior and the riskiness grade (total mean number of unsafe events per hour) and LDW. Theses correlations proved that a connection between the two variables exists, and that might be a way to improve the older population's head movements' behavior at intersections by using current in-vehicle technology.

The connection between CWS unsafe events and head movements' behavior was not trivial because CWS technology monitors driving throughout the whole driving period and counts unsafe events in a wide range of driving situations activated from 30 km/h and above. However, the IVC road scan coding in the study focuses only on intersections where drivers tend to slow down naturally according to road infrastructure. The CWS scores consisted of the number of unsafe events recorded during the driving period in relation to the drivers' travel time and as noted in the method section. These scores included a variety of driving events (such as lane deviations and risk of rear-end collisions), under a variety of road or traffic situations (such as dense traffic in urban roads or inter-urban highways). Driving at intersections is just a small part of this variety of driving situations and conditions. Similar correlations between driving performance and visual distraction were reported by other researchers (Hirayama et al., 2013; Yang et al., 2015).

Additional results of the study showed a significant increase in both the number of kilometers traveled and the number of driving hours among the older drivers at the intervention phase, when the CWS was active, compared to the pre- and post-activation phases. In old-adults, however, there has been a marked decline in driving kilometers and the number of driving hours throughout the phases. Research data from phase 1 showing that older drivers have reduced driving kilometrage and driving hours are in line with known literature that older drivers conduct “self-regulation” on their driving to reduce the likelihood of accidents. Self-regulation is defined as making adjustments in driving to accommodate for changes in cognitive, sensory, and motor capabilities, such as shorter distance travel, avoiding rush hour travel, only driving in urban settings, avoiding nighttime travel, or stopping driving in general (Ekelman et al., 2009; Meng and Siren, 2012; Svancara et al., 2020). It seems that older drivers may benefit from the presence of CWS in their vehicles, as drivers with these systems appear willing to drive more and as a result maintain their mobility option to a greater extent than drivers without the technology. It is possible that driving with immediate feedback, alerting to possible dangers on the road, may have increased their confidence to drive even at times when they had previously tried to reduce driving. In the third phase, when the feedback “crutches” were removed, there was a return to the previous driving patterns as in the first phase. On the other hand, among old-adults that do not regulate their driving yet, the intervention phase has not caused a significant change, as seen in the older drivers.

These findings, along with the findings that older drivers tended to trust the CWS as manifested by the significantly higher number of hours they drove during the intervention phase present a complex picture. On the one hand, older drivers are more confident to drive with the help of a CWS which is a good thing as it supports their independent mobility. On the other hand, the CWS is not perfect and while it improves road scanning performance in some cases it impairs other cases. Thus, the inclusion of CWS and other in-vehicle technologies should be done with cautious making sure that the benefits are greater than the cost and does not compromise safety.

## Limitations and Further Research

Since coding relied solely on head movements, if drivers depended only on their eye movements, UFOV, or wore sunglasses, an improper head movement was registered. However, the driver might have managed to road scan the area, but without considerable head movement. When considering the results, it is important to take into account that the analyses are based on a relatively small sample. Scoring on a 0–2 scale may have limited the sensitivity of this metric further. As was mentioned above, the present study compares older drivers to old-adults, aged 55–65, and maybe if the study had compared older drivers to younger drivers, the result would have been more similar to the literature. Further research is needed to better understand the right combination of immediate and retrospective feedback to maximize its impact on driving behavior. Further studies are warranted to examine the combined feedback on improving road scanning patterns of older drivers without compromising their safety.

## Conclusions and Recommendations

1. The immediate feedback of the CWS encouraged the older population to drive significantly more hours during the intervention phase and thus increased their involvement in everyday life. Being aware of the CWS's benefit can assist in making sure that older drivers continue enjoying a normal yet safe driving routine.
2. The results of this study showed that the immediate feedback provided by the CWS was partially effective in terms of participants' head movements at certain intersections (T-junctions, turns, and four-ways) but not in others (roundabout and merges). The feedback intervention was not effective in terms of reducing the number of CWS events across the study phases.
3. The CWS is currently considered a “Stand-Alone” technology (meaning only the driver receives the technological feedback), and it is not enough to be used as an educational tool for older drivers. Therefore, additional mediation or follow-up is recommended for ensuring older drivers' safety.
4. The combined feedback (immediate and retrospective) could allow drivers to receive an immediate response on unsafe driving events and “near accidents,” and get a more detailed explanation of the meanings and consequences of their impaired driving behavior later on.
5. Combining the information from CWS's alerts and IVC with a telematics system will allow fleet managers/safety organizations/driving rehabilitators to analyze the driving patterns and habits of each older driver. With this hard data, they could support and train drivers who have bad driving habits as well as reward safe drivers' behavior. Retrospective feedback can be given to vehicle fleets by an authority, such as a fleet manager, through a feedback call/driving rating compared to other drivers in the company/providing reward to those who improve.
6. In regards to elderly drivers who drive in private vehicles, driving improvement can be rewarded through providing discounts when buying insurance (Pay as you drive), or mobile feedback messages to summarize a driving period.
7. Nevertheless, when introducing such aiding technologies to the vehicles of elderly drivers, caution should be exercised because these technologies have a complex-positive and negative effect that needs to be examined in further studies.

## Data Availability Statement

The raw data supporting the conclusions of this article will be made available by the authors, without undue reservation.

## Ethics Statement

The studies involving human participants were reviewed and approved by the University of Ben-Gurion in the Negev, Israel Institutional Review Board (IRB). The patients/participants provided their written, informed consent to participate in this study. The patients/participants provided their written informed consent to participate in this study.

## Author Contributions

All authors listed have made a substantial, direct and intellectual contribution to the work, and approved it for publication.

## Conflict of Interest

The authors declare that the research was conducted in the absence of any commercial or financial relationships that could be construed as a potential conflict of interest.

## Footnotes

^1^https://drive.google.com/file/d/1uNCPIwmqysQLen7Zidj5L4j93wYscwgk/view
