# Supplementary material for: The Effects of an In-vehicle Collision Warning System on Older Drivers' On-road Head Movements at Intersections
Source: Front Psychol. 2021 Feb 19;12:596278. doi: 10.3389/fpsyg.2021.596278 (PMC7932995; doi:10.3389/fpsyg.2021.596278)
Supplement: Supplementary file 1 [file Table_1.DOCX]

Appendix A.: list of all scan demands for each intersection and driving directions

|  |  | **Scan Demands** | | | |
| --- | --- | --- | --- | --- | --- |
|  | **Intersection Type** | **Scan L** | **Scan R** | **Scan F** | **Secondary Scan** |
| 1 | Merge-R |  |  |  |  |
| 2 | Merge-L |  |  |  |  |
| 3 | Roundabout-R |  |  |  |  |
| 4 | Roundabout-L |  |  |  |  |
| 5 | Roundabout-F |  |  |  |  |
| 6 | Turn-R |  |  |  |  |
| 7 | Turn-L |  |  |  |  |
| 8 | T Junction-R |  |  |  | L |
| 9 | T Junction-L |  |  |  | R |
| 10 | Four-ways intersection-R |  |  |  | L |
| 11 | Four-ways intersection-L |  |  |  | R |
| 12 | Four-ways intersection-F |  |  |  | R+L |

Note. L=Left, R= Right, F= Forward. The study notes that coders had the option for operating personal judgment on the head scan demands as a function of the geometric structure of the intersection, its size, the number of vehicles and pedestrians in the vicinity of the intersection, while considering the degree of visibility the driver had.
